# Salmonid Jumping and Playing: Potential Cultural and Welfare Implications

**DOI:** 10.3390/ani7060042

**Published:** 2017-05-30

**Authors:** Robert M. Fagen

**Affiliations:** Ronin Institute, Montclair, NJ 07043, USA; 8sitaya@gmail.com; Tel.: +1-907-789-4608

**Keywords:** fish jumping, play behavior, pleasure in animals

## Abstract

**Simple Summary:**

Salmonids jump from the water in nature and in confinement. These behaviors are economically important and relevant to fish welfare. In net pen culture, the need to control parasitic sea lice has motivated studies of salmonid jumping behavior. Some instances of jumping in salmon may be a form of play. Indigenous and institutional science, cultural wisdom, and direct observation can aid the understanding of these behaviors.

**Abstract:**

Salmonids of several species and other fishes can jump into the air from the water. This behavior has been used in net pen culture applications to control parasitic sea lice. The reasons that salmonids jump remain a topic for speculation. Research on these behaviors has focused on Atlantic salmon in net pen culture in Northwest Europe. Jumping in salmonids is a heterogeneous behavioral category with diverse functional outcomes. Additional research is needed from broad perspectives spanning indigenous and institutional science, cultural wisdom, and ethological direct observation. In theory and in practice, it is interesting that some salmonid jumping behavior may be a form of play.

***tobu ayu no soko ni kumo yuku nagare kana***

**ayu jump**

**and beneath them flow**

**the moving clouds**

**Onitsura (1661–1738)**

## 1. Introduction

Jumping (termed leaping by some authors) is a familiar behavior in fishes (e.g., [1] (pp. 194–195), [2,3,4,5], [6] (pp. 98–99). Jumping in immature and adult salmonids is well known (e.g., [1] (pp. 194–195), [7] (p. 175), [8] (p. 136), [9] (p. 143)). It is important in practical fish culture and management, especially in the control of sea lice [10,11,12,13,14,15,16,17,18,19] and in fishway design [20,21,22,23,24]. 

The key concept of organism-environment interaction [25] is well illustrated by salmonid ecology. In salmon ecosystems, fish, forests, interconnected waterbodies extending from glaciers and icefields to the open ocean, mycorrhizal fungi, nitrogen-fixing bacteria, myriad single-celled and multicellular species, and humans are tightly and deeply interdependent [9,26,27,28,29]. The polyrhythms of life in moving fluids [30,31] are the drumbeat for this ecological dance [32].

Worldviews that recognize eco-centric values are widely acknowledged (e.g., [33]). They serve as a basis for informed critiques of the extreme reductionist views that flourished briefly in America and Western Europe during the late 20th century [34]. 

On the Northwest Coast of North America and throughout northern countries worldwide, salmonids and people have lived together for millennia [26,27,28,29,32]. The welfare of salmonids and the welfare of people are tightly linked [26,27,28,29,32]. There is a need for general theoretical treatments of salmonid culture and welfare in broad ecological contexts, as well as for practical studies that can enhance population and stock survival and sustainable harvests. Humanely ensuring survival and sustainability is equally important for salmonids and their habitats, for traditional cultures, for fishing families, and for aquaculture enterprises. 

## 2. Materials and Methods 

The materials used in this paper include traditional ecological and cultural knowledge, scientific literature, personal communications from experienced salmonid biologists, and personal experience in Alaska, Yukon, and the Pacific Northwest. The traditional ecological knowledge of Alaska, Yukon, and Pacific Northwest salmon peoples continues to inform the cultural and intellectual life of the entire region. North American ecological and environmental thought are indebted to indigenous science. The scientific study of salmon jumping is an opportunity to participate in the material and methodological convergence [35] of indigenous and Western institutional science. It also opens perspectives on broader issues of salmonid culture, welfare, and awareness. 

## 3. Results

### 3.1. Descriptive Ethology of Salmon Jumps

The ethology of these jumps is well defined and clearly differentiated from the surface rolls and oriented stream ascent leaps that salmon also perform. When an Atlantic salmon (*Salmo salar*) in a net pen [4] or a Pacific salmon (*Oncorhynchus* sp.) in its natural habitat ([1] (pp. 194–195), [7] (p. 175), [8] (p. 136)) jumps from the water, it may perform one or more lateral body flexions during its ascent to a height above the water of one or more body lengths and again during its descent. The fish frequently land on their side and make an audible splash as they reenter the water. The “characteristic leaping behaviour” of pink salmon begins with a “forward leaping motion” followed by a lateral rotation that causes the fish to fall on its side or back, and “a rapid series of jumps by the same fish often takes place” [8] (p. 136). 

### 3.2. Sea Lice, Net Pens, and Atlantic Salmon Jumps

Sea lice (*Lepeophtheirus salmonis* and *Caligus* spp.) are parasitic crustaceans (Siphonostomata: Caligidae). They represent a challenge to the net pen culture of Atlantic salmon [4,17,18,19]. An ingenious control scheme for this parasite [4] exploits the natural tendency of Atlantic salmon to jump out of the water. To facilitate the control of sea lice, net pen fish are induced to jump through a surface film that contains a chemical treatment that kills the lice. The natural frequency of spontaneous jumps in Atlantic salmon is low (~1 jump per individual per 2 h [4]). For this reason, managers have studied salmon jumping. Their goal is to identify factors that can be manipulated to increase the jumping frequency and, therefore, the effectiveness of the treatment regime. 

### 3.3. Why do Salmonids Jump?

Away from work and sometimes even at work, fisheries professionals and recreational fishers often discuss the possible reasons that salmon jump. As one reviewer noted, biologists frequently go salmon fishing after work, and the highlight of most fishing stories back in camp is the vigor with which a hooked salmon leapt. 

Jumping may serve to loosen egg skeins; dislodge ectoparasites; evade predators; reduce perceived crowding; communicate with conspecifics; avoid supersaturated, turbulent, hyposaline and/or turbid water; and/or refill the swim bladder with air. All of these functional hypotheses are open to discussion and investigation. For example, F.A. Beach [36] proposed that jumping functions to remove ectoparasites because he opposed the idea that fish jumping might sometimes represent play behavior. Burghardt [3] more recently marshaled new information and deployed clearer reasoning to criticize this argument. 

In the Tlingit culture of southeast Alaska, tradition offers specific insights into salmon jumping. An indigenous perspective mentioned to non-Natives by elders and culture-bearers is that a salmon jumps for the same reason that a person stands up in a boat to better see the surrounding waters and land. This hypothesis, based on indigenous science, that jumping in Pacific salmon can function to facilitate above-water visual orientation in space could productively be tested in field and experimental trials. In the Tlingit language [37,38], one of the meanings of the verb root √TAAN is “to jump (fish)” [38]. It would be interesting to consider other traditional cultures both on the Pacific Coast and worldwide to find traditions and linguistic terms that applied to salmon jumping. 

It is also possible that some instances of salmon jumping are best categorized as play behavior. Dennis Dobson, a veteran Pacific Northwest fishing guide and outdoor journalist, argues that jumping in the fish he has observed over decades in the wild can be plausibly interpreted as play [39]. His arguments rely on extensive direct observations and experience, and his descriptions appear to fall within the scope of current scientific definitions of play. Dobson’s interpretation, like other plausible hypotheses about salmon jumping, seems scientifically credible. The method of multiple working hypotheses [40] furnishes a fruitful methodological and philosophical basis for testing hypotheses about salmon jumping. 

## 4. Discussion

That play behavior is by no means restricted to warm-blooded vertebrates has become increasingly evident. Play (including several instances of jumping, though not in salmonids) is now well documented in fishes [3,6] (p. 94–98). Jennifer Nielsen [3] (p. 148) observed a possible instance of movement play (not involving jumping) resembling adult redd-digging behavior in juvenile coho salmon (*Oncorhynchus kisutch*). In fact, vertical leaps are a paradigmatic instance of play in nonhuman species [41]. As one referee informatively stated, “jumping for joy” is a timeless expression: Duke Ellington not surprisingly “jumped for joy” when he left Cotton Isle. So did Kingfish (Bob Weir and company) when they were “coming back to ‘Frisco”. Baby Roo, Tigger, and Pooh jumped for joy often.

For additional discussions of animal play behavior, see, e.g., [3,41,42,43]. 

Burghardt [3,44,45] proposed an open, inclusive definition of play. His definition has a sound basis in classical continental European ethology (e.g., [46]) and has been well received by ethologists and zoologists. Furthermore, Burghardt, Dinets and colleagues [47] have substantially enlarged the empirical basis for the belief that play behavior occurs in fishes. In turn, Marc Bekoff [48,49] has placed these and other observations in a broad ethical context relating play to animal awareness, suffering, and pleasure, and to natural principles of fairness in animal communication and sociality. (See also [50,51].)

Current views of the deep ecology of salmonids (e.g., [9]) cite holistic perspectives. Tlingit people might well recognize echoes of their own Raven ecology in these latter-day views. John Muir first encountered this traditional indigenous and ancient science of natural balance and harmony in nature when he visited Tlingit elders Daanaawaak and Lunaat’ in Alaska’s Chilkat-Chilkoot country. During his few days’ visit with them and their people in a Tlingit village near present-day Haines, Alaska, John Muir learned the ecology lesson he would never forget [52,53]. Muir’s ecological reeducation in Tlingit country would eventually influence society. Muir’s ecological ideas and environmental activism grew organically from indigenous Tlingit and additional Native Alaskan and First Nations sources, spurring later mainstream North American scientific ecology and philosophical and practical environmentalism. For example [54,55,56], environmental ethicist and freshwater salmonid biologist J.J. Piccolo spent years with Native Americans and Alaskan Natives as a fisheries scientist on their traditional lands. He later insightfully integrated this experience with the work of scholars such as Aldo Leopold and Holmes Rolston, who had also been influenced by the Daanaawaak-Lunaat’-Muir tradition.

Alaska fisheries researcher Nicholas F. Hughes contributed paradigmatic insights into the ecology of salmonids in moving water. He provided a general framework within which ecological interactions among fish in flowing water might be profitably explored [57,58,59]. Reasoning based on this framework has spurred a fecund theoretical perspective termed Net Energy Intake Theory. The theory has shed light on several aspects of salmonid cognitive/social behavior, including social learning for foraging and recognition of familiar partners [60,61]. In immature salmon, the perceptual, cognitive, and sensory-locomotor salience of water streaming from hoses and pipes and splashing from buckets is a developmental and ecological puzzle. Clearly, however, considering the pervasiveness of organism-environment interactions shaped by moving water, the development of behavioral flexibility and cognitive-motor strengths and skills involving the physics and dynamics of life in moving fluids could be important for behavioral and cognitive development in salmonids beginning in early ontogeny. Indeed, species, stock, population, or strain differences having ecological, aquacultural, and welfare implications could be investigated, as each salmonid species, stock, or strain can experience different contrasting and varying flow regimes throughout its life cycle (e.g., [1,8,62]). In several mammalian species, play increases subsequent survival or components of survival such as physical capacity [63,64,65,66,67]. Whether play confers such benefits in nonmammalian taxa is not yet known. Subjectively, a stream of water might well represent an exciting sensorimotor and aesthetic property of the environment to a young salmonid (in the sense of Darwinist aesthetics [68]) as well as an ecologically salient feature that might stimulate it to leap repeatedly with no immediate ulterior purpose. 

## 5. Conclusions

Jumping play in salmonids is of potential interest as a measure of well-being and as a potential component of survival and fitness. Whether salmonid play necessarily implies the ability to experience pleasure and to suffer and/or constitutes indirect evidence for consciousness may be a challenging question for the future.
